# Disorders of Sex Development in Individuals Harbouring *MAMLD1* Variants: WES and Interactome Evidence of Oligogenic Inheritance

**DOI:** 10.3389/fendo.2020.582516

**Published:** 2020-12-23

**Authors:** Lele Li, Fenqi Gao, Lijun Fan, Chang Su, Xuejun Liang, ChunXiu Gong

**Affiliations:** Department of Endocrinology, Genetics, Metabolism and Adolescent Medicine, Beijing Children’s Hospital, The Capital Medical University, National Center for Children’s Health, Beijing, China

**Keywords:** disorders of sexual development, MAMLD1, oligogenic inheritance, hypospadias, whole exome sequencing

## Abstract

Mastermind-like domain-containing 1 (MAMLD1) has been shown to play an important role in the process of sexual development and is associated with 46,XY disorders of sex development (DSDs). However, the causative role of *MAMLD1* variations in DSDs remains disputable. In this study, we have described a clinical series on children from unrelated families with 46,XY DSD harbouring *MAMLD1* variants. Whole exome sequencing (WES) was performed for each patient. WES data were filtered using common tools and disease customisation algorithms, including comparison against lists of known and candidate MAMLD1-related and DSD-related genes. Lastly, we investigated the hypothesis that MAMLD1-related DSD may follow an oligogenic mode of inheritance. Forty-three potentially deleterious/candidate variants of 18 genes (*RET*, *CDH23*, *MYO7A*, *NOTCH2*, *MAML1*, *MAML2*, *CYP1A1*, *WNT9B*, *GLI2*, *GLI3*, *MAML3*, *WNT9A*, *FRAS1*, *PIK3R3*, *FREM2*, *PTPN11*, *EVC*, and *FLNA*) were identified, which may have contributed to the patient phenotypes. *MYO7A* was the most commonly identified gene. Specific gene combinations were also identified. In the interactome analysis, *MAMLD1* exhibited direct connection with *MAML1*/*2*/*3* and *NOTCH1*/*2*. Through *NOTCH1/2*, the following eight genes were shown to be associated with *MAMLD1*:*WNT9A/9B*, *GLI2/3*, *RET*, *FLNA*, *PTPN11*, and *EYA1*. Our findings provide further evidence that individuals with MAMLD1-related 46,XY DSD could carry two or more variants of known DSD-related genes, and the phenotypic outcome of affected individuals might be determined by multiple genes.

## Introduction

Disorders of sex development (DSDs) comprise a group of congenital diseases associated with the atypical development of internal and external genital structures. These conditions may be related to genetic variation, developmental programming, and hormone expression (1). Our understanding of DSDs has evolved significantly over the past several decades owing to the extensive research conducted on mammalian sex development and the genetic mechanisms underlying DSDs (2–5). Various underlying causes, such as mutations in the genes encoding proteins associated with sex determination and development as well as genital development, have been described (5). However, genotype-phenotype correlations are difficult to evaluate owing to the high phenotypic and genotypic diversity among individuals.

Mastermind-like domain-containing 1 gene (*MAMLD1*), also known as chromosome X open reading frame 6 (*CXorf6*) or *F18* (online Mendelian inheritance in man (OMIM)# 300120), was first reported in two cases of myotubular myopathy and male hypogenitalism (6, 7). It was identified as a suitable candidate gene in patients with 46,XY DSD and was shown to be expressed in foetal Leydig cells at a time point close to the critical period for sex development (8, 9). In mouse Leydig tumour cells, the transient knockdown of *Mamld1* mRNA expression led to a significant reduction in testosterone (T) production (10). *MAMLD1* contains the target sequence of steroidogenic factor (SF-1), which is a modulator of gene transcription involved in testicular differentiation (11, 12). MAMLD1 transactivates the non-canonical Notch-targeted *Hes* 3 promoter (8, 12). Hes3 regulates cell differentiation and proliferation during embryonic development (13). Therefore, MAMLD1 appears to play an important role during sex development and is associated with 46,XY DSD.

To date, approximately 30 *MAMLD1* sequence variations have been identified in 46,XY DSD patients and recorded in the human gene mutation database (14). Disease-causing *MAMLD1* variants can carry nonsense, missense, or frameshift mutations; insertions; or deletions, and these may even include complex variants (14). They are found to be scattered throughout the gene sequence and are not restricted to significant hotspots, owing to which the genotype-phenotype correlations remain obscure (14). Patients with 46,XY DSDs share a wide variety of phenotypic features (8, 9). The most significant phenotypic feature observed is hypospadias. Other phenotypes include cryptorchidism, micropenis, complete female external genitalia, and primary amenorrhea (14–17).

However, even after investigation of the condition, the causative role of *MAMLD1* variations in DSDs remains disputable for several reasons. First, some *MAMLD1* variants (P359S, V505A, and N662S) have also been identified in normal individuals (9, 15, 16), while others (P359S and Q580R) have not been detected in all affected patients from a single family (9). Second, several *MAMLD1* variations have been confirmed to be associated with wild-type activity in functional studies (15, 18), and the animal experiments have shown that *Mamld1*-KO male mice present with normal genitalia and reproduction (19). Therefore, the role of MAMLD1 in sex development requires further elucidation; the broad spectrum of phenotypes indicates the presence of various modifying factors, such that a single pathogenic variant may neither be necessary nor sufficient for pathogenesis.

Our understanding of the genetic architecture of sex development-related inherited disorders has increased considerably over the past few years. Initial discoveries pertained to the identification of genes encoding proteins associated with disorders with Mendelian (monogenic) inheritance. In recent years, gene discovery efforts have evolved to consider more complex inheritance patterns, such as oligogenic inheritance, in which the accumulation of inherited low-penetrance variants in multiple genes contributes to the disorder (20). Oligogenic inheritance has been noted in several disorders, such as in congenital hypogonadotropic hypogonadism (21), inherited cardiac disorders (20), and NR5A1-related DSDs (22), using high-throughput sequencing (HTS).

In the present study, we investigated the hypothesis that MAMLD1-related DSDs may follow an oligogenic mode of inheritance. Whole exome sequencing (WES) was performed for ten subjects with 46,XY DSD harbouring *MAMLD1* variants. WES data were filtered using common tools and a disease-tailored algorithm including lists of MAMLD1-related and DSD-related known and candidate genes designed by Fluck et al. (23). Using this method, we attempted to provide evidence that phenotypic outcomes may be determined by multiple genes.

## Materials and Methods

### Subjects

Patients ranged in age from 2 months to 14 years and had been admitted to Beijing Children’s Hospital in the past 5 years. Ten patients (1–10) from unrelated families, the members of which were confirmed to harbour mutations in *MAMLD1*, were recruited in the study. Each of the patients were assigned the same number as in our previously reported study (14). Patients with abnormal liver or kidney function, or those with systemic diseases that affect physical development, were excluded.

**Figure 1:**
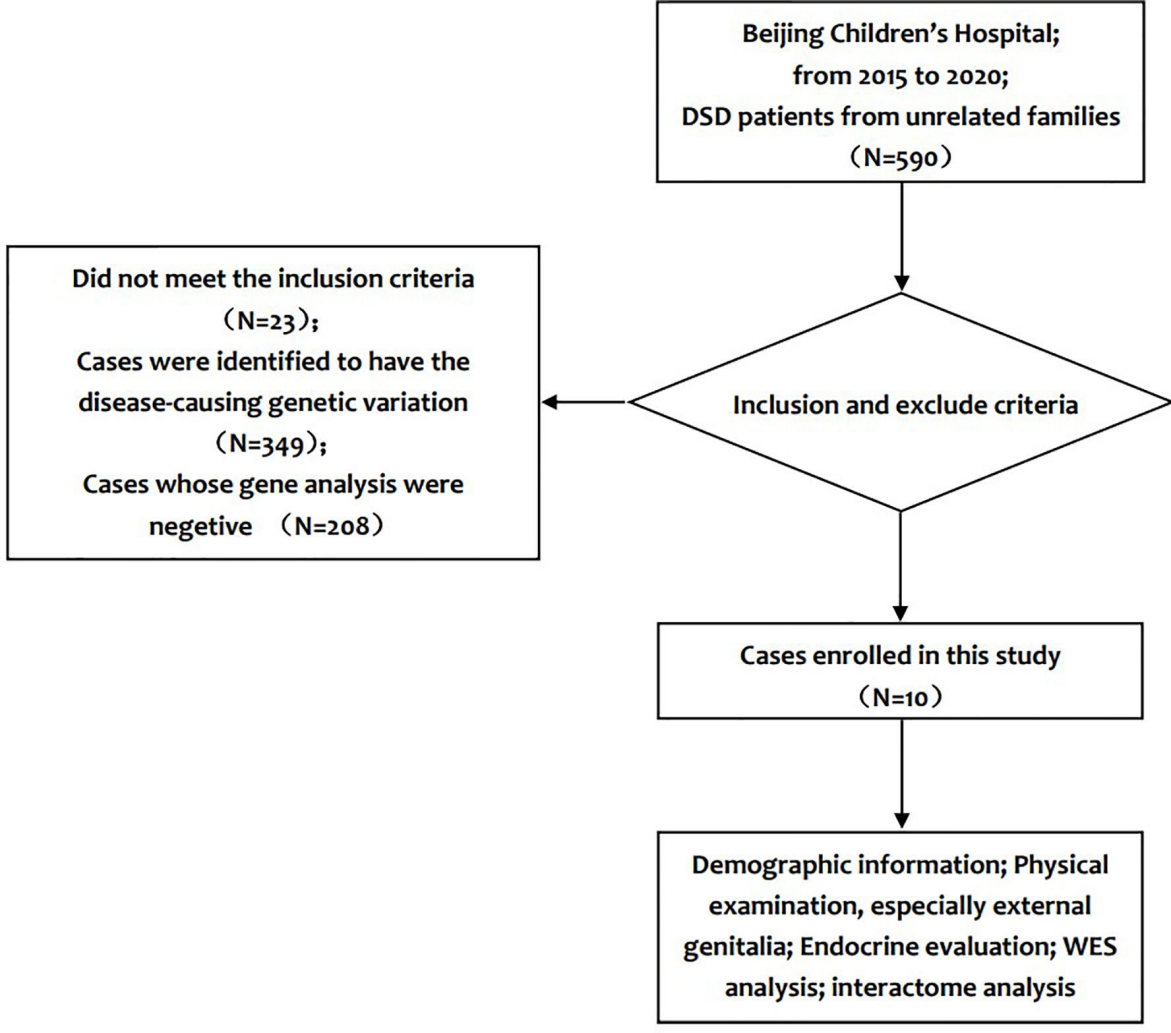
Flow chart of the study design.

**Figure 2:**
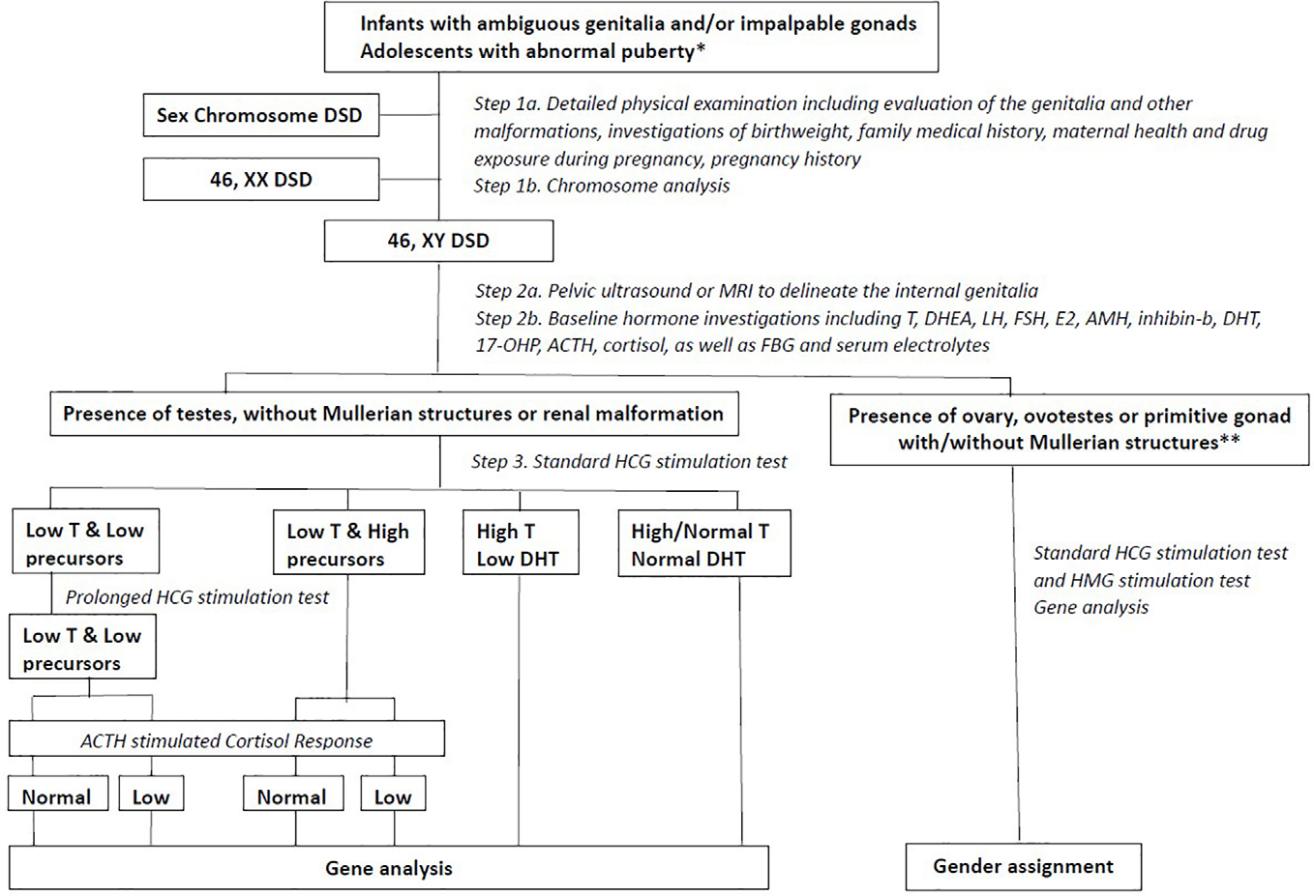
The clinical evaluation algorithm of DSD patients. * abnormal puberty indicating that a girl with primary amenorrhoea with or without breast development, a girl who virilizes at puberty or a boy with pubertal delay; DSD, Disorders of Sex Development; FSH, Follicle Stimulating Hormone; LH, Luteinizing Hormone; T, Testosterone; DHEA, Dehydroepiandrosterone; E2, Estradiol; AMH, Anti-Mullerian Hormone; DHT, Dihydrotestosterone; 17-OHP, 17-OH-Progesterone; ACTH, Adrenocorticotrophic Hormone; FBG, Fast Blood Glucose; HCG, Human Chorionic Gonadotropin; HMG, Human Menopausal Gonadotropin; CHH, Congenital Hypogonadotropic Hypogonadism; Dysg, Gonadal Dysgenesis.

Clinical information was obtained from the medical record of each patient. Two experienced paediatric endocrinologists performed physical examinations and assessments. The information included, but was not limited to, age at visit, social gender, chief complaint, family history, bone age, birth length, birth weight, gestational age, history of gestation, penile length, testis size, testis position, urethral meatus, scrotal appearance, electrolyte levels, and liver and kidney function. Hormone investigations involved measurement of the levels of luteinizing hormone, follicle-stimulating hormone, anti-Müllerian hormone, inhibin-B, testosterone, adrenocorticotropic hormone, cortisol, 17-hydroxyprogesterone, and dehydroepiandrosterone sulphate. A flow chart of the study design is presented in **Figure 1**. The clinical evaluation algorithm of patients with DSD is presented in **Figure 2**.

### Molecular Analysis

WES was performed for all patients. Genomic DNA was extracted from the peripheral blood samples of probands and their parents, and was sent to Chigene Translational Medicine Research Center Co., Ltd (Beijing Kangso Medical Laboratory Zhongguancun Huakang Gene Institute) for commercial sequencing. Putative candidate variants were confirmed using Sanger sequencing.

### Bioinformatics Analysis

WES data were filtered using a disease-tailored list of MAMLD1-related and DSD-related known and candidate genes (N=606), similar to the algorithm previously designed by Camats et al. (22, 23). A project-specific filter for DSD-related and MAMLD1-related genes was generated by conducting a search in published literature and databases. The DSD-related gene list included genes with (potentially) deleterious variants reported in patients with 46,XY and 46,XX DSDs; genes with (potentially) disease-causing variants reported in syndromic patients with involvement of sex development; genes “related” to DSD in KO/mutant animal models (mice and rats); and overexpressed, upregulated, or downregulated genes in rodent embryonic gonadal cells. The following bioinformatics software tools were used for the interpretation and classification of variants: InterVar (http://wintervar.wglab.org/; clinical interpretation of genetic variants using the ACMG/AMP 2015 guideline), VarSome (The Human Genomics Search Engine; https://varsome.com/), ClinVar (https://www.ncbi.nlm.nih.gov/clinvar/), and Alamut Visual 2.11 (https://www.interactive-biosoftware.com/es/alamut-visual/).

After annotation, variant analysis was performed according to the following steps. A) WES data of each patient were first filtered using the MAMLD1- and DSD-related known and candidate gene lists; B) Variants with minor allele frequency (MAF) <0.05, or those undetected in gnomAD, 1000 Genomes (China), and ExAC (East Asia), were retained; C) Variants that were considered irrelevant for our study, including 1) variants detected in more than two patients, 2) variants in repeat regions, 3) variants in genes or gene regions with high variability (MAF >0.05), and 4) variants with low coverage and/or low quality, were discarded.

A search was performed for reported (potentially) disease-causing variants using the Human Gene Mutation Database (HGMD^®^ Professional 2018.2, http://www.biobase-international.com/product/hgmd; Biobase) and dbSNP (http://www.ncbi.nlm.nih.gov/snp/). The search tool for the retrieval of interacting genes/proteins (STRING, http://string-db.org/) was used to analyse interactions within gene carriers of notable variants (DSD-related and/or MAMLD1-related). A medium confidence of 0.400 was noted. STRING data were extracted from known interactions (curated databases, experimentally determined interactions), predicted interactions (gene neighbourhood, gene fusions, gene co-occurrence), and other inferred evidences such as text mining, co-expression, and protein homology.

### Ethics Approval

The project was approved by the Institutional Medical Ethics Review Board of Beijing Children’s Hospital (ID: 2019-k-156). Written informed consent was obtained from all patients or their legal guardians. This study was conducted in accordance with the principles of the Declaration of Helsinki.

## Results

### Clinical Features

**Figure 3:**
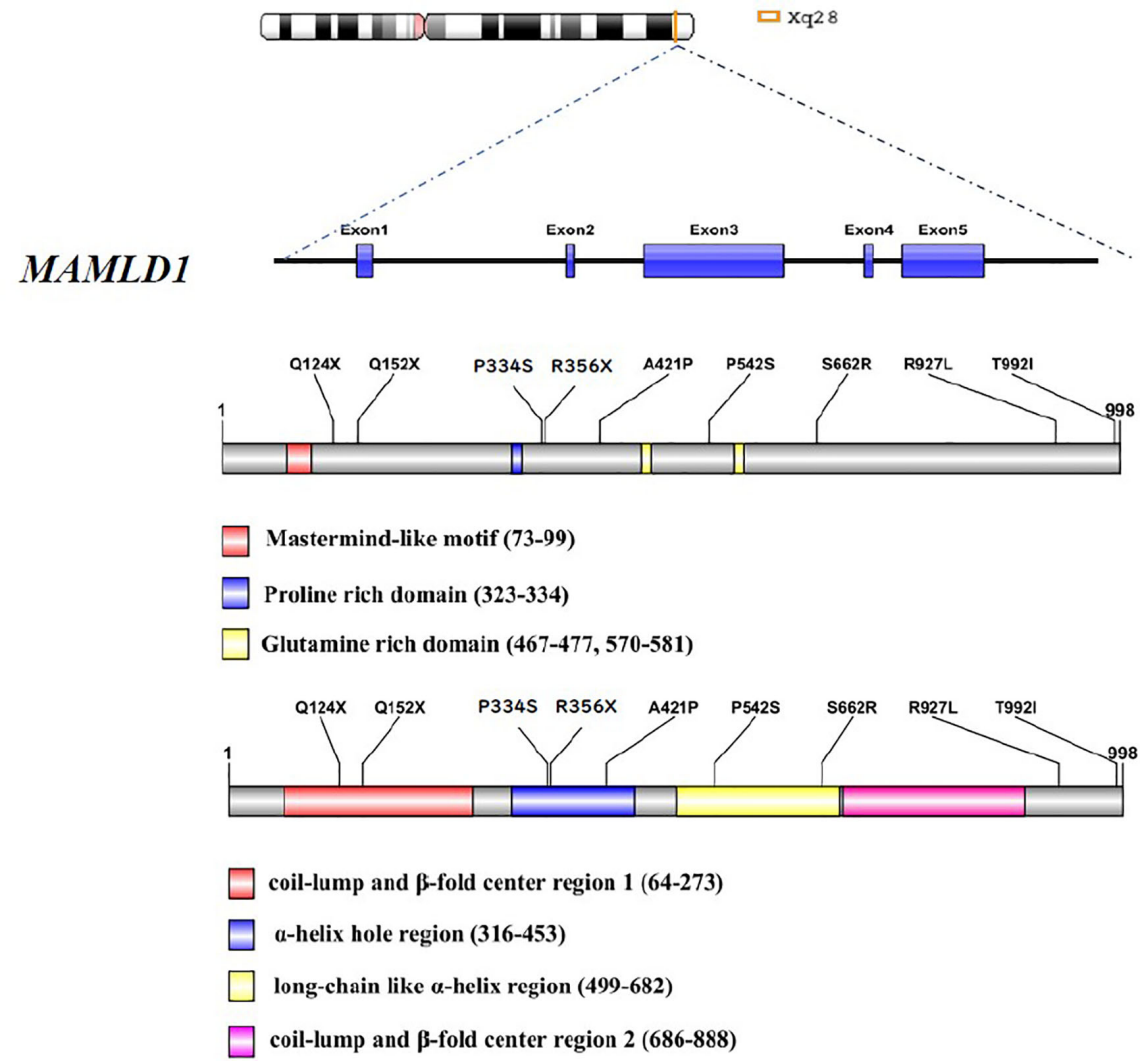
Detailed variation information of functional domains of the nine *MAMLD1* variants.

The clinical features, hormone profiles, and molecular results of the ten Chinese patients harbouring *MAMLD1* mutations are summarised in a previously published article (14). The age at visit of each subjected was under 3 years. The salient phenotypic feature was hypospadias (8/10); other phenotypic features included cryptorchidism (3/10) and micropenis (7/10). Serum T, luteinizing hormone, and follicle-stimulating hormone levels were sufficiently high in patients #3 and #6, who were in the mini-puberty period. An adequate response of T levels to human chorionic gonadotropin (hCG) stimulation was observed in patients #1–3, #6, and #8. Overall, nine genetic variants were identified, including six missense variations (p.P334S, p.S662R, p.A421P, p.T992I, p.P542S, and p.R927L) and three nonsense variations (p.R356X, p.Q152X, and p.Q124X). All patients had inherited the variants from their mothers. Detailed information on the functional domains is presented in **Figure 3**.

### Identification of an Oligogenic DSD Aetiology in Individuals With 46,XY DSD Harbouring *MAMLD1* Variants

Forty-three potentially deleterious/candidate variants of 18 genes (*RET*, *CDH23*, *MYO7A*, *NOTCH2*, *MAML1*, *MAML2*, *CYP1A1*, *WNT9B*, *GLI2*, *GLI3*, *MAML3*, *WNT9A*, *FRAS1*, *PIK3R3*, *FREM2*, *PTPN11*, *EVC*, and *FLNA*) were identified in the ten hemizygous MAMLD1 patients (**Table 1**). These variants may have contributed to the phenotype of each patient.

**Table 1:** Identified genes and variants per patient after specific filtering.

Patient	Gene	Locus	Type	HGVSc,HGVSp	dbSNP ID	Mutation source	MAF Value			ACMG classification
							GnomAD	1000Genomes (China)	ExAC (East Asian)	
1	MAMLD1	Xq28	miss	c.1986C>G, p.S662R		mother				VUS[(PM2,BP1)]
1	RET	10q11.2	syn	c.3120G>C, p.L1040L		mother				VUS(PM2,BP7)
1	CDH23	10q22.1		c.6712+37C>T	rs750966744	father	0.000032		0.0003	VUS(PM2)
1	MYO7A	11q13.5		c.3504-39T>C	rs192929996	mother	0.0013	0.019	0.017	VUS(BS1)
1	MAML1	5q35	syn	c.1893G>A, p.Q631Q		Denove				VUS(PM2,BP7)
2	MAMLD1	Xq28	non	c.1066C>T, p.R356X	rs782347827	mother			0	P(PVS1,PM2,PP3)
2	WNT9B	17q21		c.*158C>T	rs1233082195	father	0.000008			VUS(PM2,PP5)
2	NOTCH2	1p13-p11		c.2600-24C>A	rs149097658	mother	0.0034	0.024	0.027	VUS(BS)
2	GLI2	2q14		c.845+10G>A	rs199673018	father	0.00024	0	0.0005	VUS
2	GLI3	7p13		c.1028+15G>A	rs116842918	father	0.0014	0.012	0.017	LB(BS1,BP6)
3	MAMLD1	Xq28	non	c.1066C>T, p.R356X	rs782347827	mother			0	P(PVS1,PM2,PP3)
3	MYO7A	11q13.5		c.1554+22C>T		mother	0.0006	0.0024		VUS(PM2,BP4)
3	MYO7A	11q13.5	miss	c.3124T>G, p.W1042G		mother				VUS(PM1,PM2,PP3)
4	MAMLD1	Xq28	miss	c.1261G>C, p.A421P		mother				VUS(PM2,PP3,BP1)
4	MAML3	4q28	miss	c.1612A>G, p.M538V	rs148778901	mother	0.0074	0.0072	0.0078	LB (PP3,BS1)
4	GLI3	7p13	miss	c.1843A>T, p.T615S	rs200913720	mother	0.0006	0.0024	0.0005	VUS (PP3,BP6)
4	GLI3	7p13	miss	c.674C>T, p.T225I	rs753769482	mother			0.001	VUS (PM2,PP3)
5	MAMLD1	Xq28	non	c.454C>T, p.Q152X		mother				P(PVS1,PM2,PP3)
5	RET	10q11.2	miss	c.1465G>A, p.D489N	rs9282834	mother	0.0296	0.014	0.028	LB (BS1,BP6,BP4)
5	NOTCH2	1p13-p11		c.2600-24C>A	rs149097658	father	0.0191	0.024	0.027	VUS (BS1)
5	GLI2	2q14	syn	c.4611C>T, p.L1537L	rs1335405295	father				VUS (PM2,BP7)
6	MAMLD1	Xq28	miss	c.1624C>T, p.P542S	rs146443503	mother	0.0015	0.016	0.021	LB (BS1,BP1)
6	MYO7A	11q13.5		c.6051+9C>T	rs747742075	father	0.0018			VUS (BS4)
6	CYP1A1	15q24.1	miss	c.518C>G, p.T173R	rs28399427	father	0.008	0.0072	0.0094	VUS (BP4)
6	WNT9A	1q42		c.353-30G>A	rs141239747	mother	0.0314	0.039	0.028	B (BA1,BS1,BS2)
6	FRAS1	4q21.21	syn	c.10930C>T, p.L3644L	rs183729151	father	0.0025	0.0048	0.0022	LB (BS4,BP7)
6	GLI3	7p13	syn	c.3015C>T, p.A1005A	rs200965852	father	0.0173	0.024	0.019	LB (BS1,BP6,BP7)
7	MAMLD1	Xq28	miss	c.1000C>T, p. P334S	rs41313406	mother	0.08	0.0031	0.0002	LP(PM1,PM2,PM5,PP3)
7	CYP1A1	15q24.1	syn	c.927C>T, p.N309N	rs368742906	unknown	0.0031	0.0024	0.0032	VUS (PM2,BP7,BP4)
7	PIK3R3	1p34.1	miss	c.290A>G, p.Q97R	rs1225342856	unknown				VUS (PM1,PM2)
8	MAMLD1	Xq28	non	c.370C>T, p.Q124X		mother				P(PVS1,PM2)
8	CDH23	10q22.1	miss	c.1282G>A, p.D428N	rs188376296	father	0.0031	0.0048	0.0066	VUS (PP5,PP3,BS4)
8	MYO7A	11q13.5	miss	c.1133G>A, p.R378H	rs397516282	father				LP (PM2,PM5,PP3,BS4)
8	MYO7A	11q13.5		c.4152+15A>G	rs1033447071	father				VUS (PM2,BS4)
8	FREM2	13q13.3	miss	c.4916G>A, p.R1639K	rs77886481	mother	0.0197	0.014	0.022	VUS (BS1)
8	FRAS1	4q21.21		c.7372-24A>G	rs78365404	mother	0.013	0.0096	0.019	B (BS1,BS2,BS4)
8	FRAS1	4q21.21	miss	c.8493C>G, p.F2831L	rs774409872	mother	0.0019	–	–	VUS (BS4)
9	MAMLD1	Xq28	miss	c.2975C>T, p.T992I		mother				VUS(PM2,BP1,BP4)
9	CDH23	10q22.1		c.9077+7C>T	rs76114420	mother	0.0259	0.022	0.018	LB (BS1,BP6)
9	WNT9B	17q21	syn	c.1059C>T, p.Y353Y	rs537242221	mother	0.0006	0.0024	0	VUS (BP7)
9	MAML3	4q28	miss	c.2969C>T, p.P990L	rs185593153	father	0.0006	0.0048	0.0011	VUS
9	FRAS1	4q21.21		c.5857-434C>T		mother				VUS (PM2,BS4)
10	MAMLD1	Xq28	miss	c.2780C>T, p.R927L	rs782511956	mother				LB (PM2,BP1,BP4)
10	CDH23	10q22.1		c.4210-16C>A	rs775928557	father				VUS (PM2)
10	CDH23	10q22.1		c.9077+7C>T	rs76114420	mother	0.0259	0.022	0.018	LB (BS1,BP6)
10	MYO7A	11q13.5	syn	c.324C>T, p.Y108Y	rs116892396	mother	0.0161	0.026	0.021	LB (BS1,BP6,BP7)
10	MAML2	11q21	miss	c.385G>A, p.D129N	rs892433964	mother	0.0012			VUS (PP3)
10	PTPN11	12q24		c.1448-38G>T		mother				VUS (PM2)
10	NOTCH2	1p13-p11	syn	c.6783G>A, p.E2261E	rs759118563	mother			0.0001	VUS (PM2,BP7)
10	PIK3R3	1p34.1	miss	c.950A>G, p.N317S	rs114180250	father	0.0129	0.019	0.013	VUS (BS1)
10	EVC	4p16		c.1316-20G>T	rs773461223	mother				VUS (PM2)
10	EVC	4p16		c.1564-6C>T	rs188245524	mother	0.0012	0.0024	0.0038	VUS (BP6,BP4)
10	FRAS1	4q21.21	miss	c.6569C>T, p.S2190F	rs200166354	father	0.0031	0.0024	0.0033	VUS (BS4)
10	FLNA	Xq28	syn	c.3756G>A, p.A1252A	rs186619828	mother	0	0	0	VUS (BP6)

ACMG, the American College of Medical Genetics and Genomics; MAF, Minor Allele Frequency; VUS, Uncertain Significance; P, Pathogenic; B, Benign; LB, Likely Benign; LP, Likely Pathogenic.

Among the eighteen genes, four have been previously identified in patients with hypospadias (*CYP1A1*, *FLNA*, *GLI3*, and *GLI2*), three have been reported to be associated with cryptorchidism (*FLNA*, *RET*, and *PTPN11*), and one has been identified in patients with micropenis (*EVC*). In addition, eight genes were identified in patients with DSDs (*FRAS1*, *FREM2*, and *NOTCH2*) and/or were reported to be associated with other syndromes combined with DSDs (*CYP1A1*, *EVC*, *FRAS1*, *PTPN11*, and *RET*). Fourteen genes were previously shown to be associated with sexual or gonadal development (*CDH23*, *EVC, FLNA*, *FRAS1*, *FREM2*, *GLI2*, *GLI3*, *MAML3*, *MYO7A*, *NOTCH2*, *PIK3R3*, *RET, WNT9A*, and *WNT9B*). Based on information from OMIM, all patients, except patient #7, presented at least one variant in a gene with autosomal dominant (AD) inheritance (*GLI2*, *FLNA*, *GLI3*, *NOTCH2*, *PTPN11*, and *RET*); the other genes (*CDH23* and *MYO7A*) exhibited either AD or autosomal recessive (AR) inheritance. *FLNA* undergoes X-linked recessive inheritance, while *CYP1A1, FRAS1, FREM2*, and *EVC* are known to undergo AR inheritance.

*MYO7A* was the most commonly identified gene. Seven *MYO7A* variants were identified in five patients (patients 1, 3, 6, 8, and 10); five *CDH23* variants were identified in five patients (patient 1, 8, 9, and 10); five *FRAS1* variants were identified in four patients (patient 6, 8, 9, and 10); four *GLI3* variants were identified in three patients (patient 2, 4, and 6); *NOTCH2* variants were identified in three patients (patient 2, 5, and 10). Variants of the following genes were detected in any two patients: *CYP1A1* variants in patients 6 and 7, *PIK3R3* variants in patients 7 and 10, *RET* variants in patients 1 and 5, *MAML3* variants in patients 4 and 9, *WNT9B* variants in patients 2 and 9, and *GLI2* variants in patients 2 and 5.

Patient 1 carried four variants among four genes: *RET*, *CDH23*, *MYO7A*, and *MAML1*. Patient 2 carried four variants among four genes: *WNT9B*, *NOTCH2*, *GLI2*, and *GLI3*. Patient 3 carried two variants of *MYO7A*. Patient 4 carried three variants in *MAML3* and *GLI3*. Patient 5 carried three variants among three genes: *RET*, *NOTCH2*, and *GLI2*. Patient 6 carried five variants among five genes: *MYO7A*, *CYP1A1*, *WNT9A*, *FRAS1*, and *GLI3*. Patient 7 carried two variants among two genes: *CYP1A1* and *PIK3R3*. Patient 8 carried six variants among four genes: *CDH23*, *MYO7A*, *FREM2*, and *FRAS1*. Patient 9 carried four variants among four genes: *CDH23*, *WNT9B*, *MAML3*, and *FRAS1*. Patient 10 carried 11 variants among nine genes: *CDH23*, *MYO7A*, *MAML2*, *PTPN11*, *NOTCH2*, *PIK3R3*, *EVC*, *FRAS1*, and *FLNA.* The details of these variants are summarised in **Table 1**.

In addition, patients 1, 8, and 10 presented combination variants of two genes: *MYO7A-CDH23*. Patients 2 and 5 both presented combination variants of two genes: *NOTCH2-GLI2*; patients 6 and 8 both presented combination variants of two genes: *MYO7A-FRAS1*.

### Interactome Analysis of the Identified DSD- and MAMLD1-Related Genes

**Figure 4:**
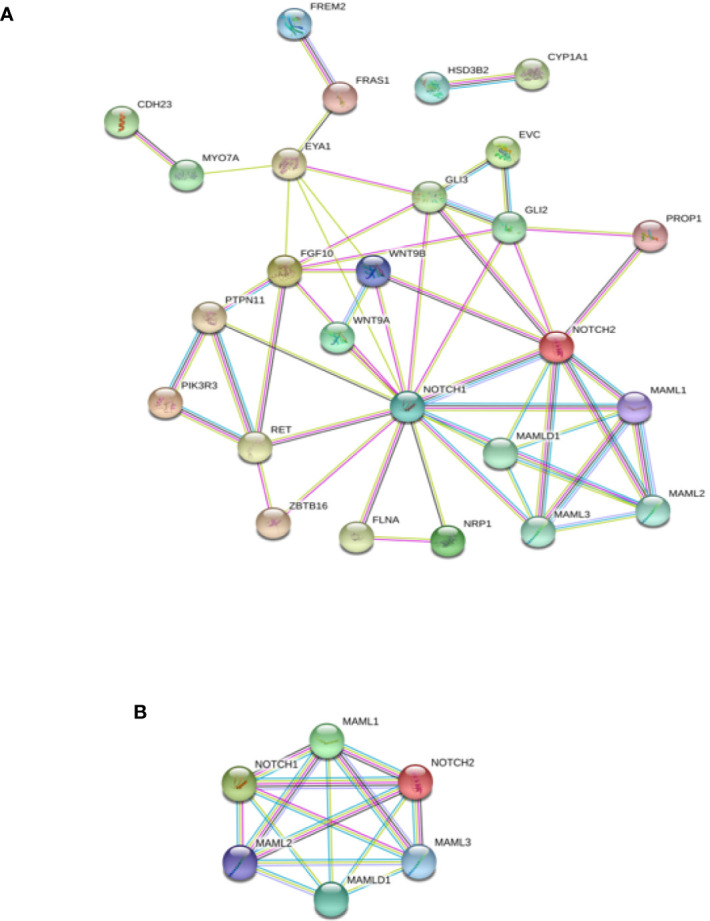
Interaction network of DSD- and MAMLD1-related genes identified in DSD individuals harbouring genetic variants in *MAMLD1*. **(A)** The scheme depicts an overview of detected genes and their interrelationship. **(B)** Core network of the detected genes and their interrelationship. Filled nodes show proteins with known or predicted 3D structure. Empty nodes depict proteins with unknown 3D structure. Candidate genes are underlined. Known interactions correspond to curated databases (turquoise lines) and experimentally determined interactions (pink lines). Predicted interactions correspond to gene neighbourhood (green lines), gene fusions (red lines) and gene co-occurrence (blue lines). Other interactions correspond to text mining (yellow lines), co-expression (black lines) and protein homology (violet lines).

Interactome analysis was performed for the identification of DSD-related genes using bioinformatics software to assess possible gene–protein interactions. The network including all genes identified is presented in **Figure 4A**. The core network constructed using the Cytoscape Molecular COmplex DEtection (MCODE) software is shown in **Figure 4B**. Overall, a connection was detected among the 18 genes and we report that *MAMLD1* is directly connected to *MAML1/2/3* and *NOTCH1/2*. Through *NOTCH*, eight genes (*WNT9A/9B*, *GLI2/3*, *RET*, *FLNA*, *PTPN11*, and *EYA1*) were associated with *MAMLD1*. Some of these genes also acted as central nodes for further connections; for example, *GLI3* for *EVC*, *FGF10*, *GLI2*, *NOTCH1/2*, and *EYA1*; *RET* for *ZBTB16*, *FGF10*, *PIK3R3*, *PTPN11*, and *NOTCH1*; *EYA1* for *FRAS1*, *MYO7A*, *FGF10*, *NOTCH1*, *WNT9B*, and *GLI3*; and *FGF10* for *EYA1*, *GLI2/3*, *NOTCH1*, *PTPN11*, and *RET*. In addition, the isolated gene couple that was revealed in our analysis was *CYP1A1-HSD3B2*.

**Figure 5:**
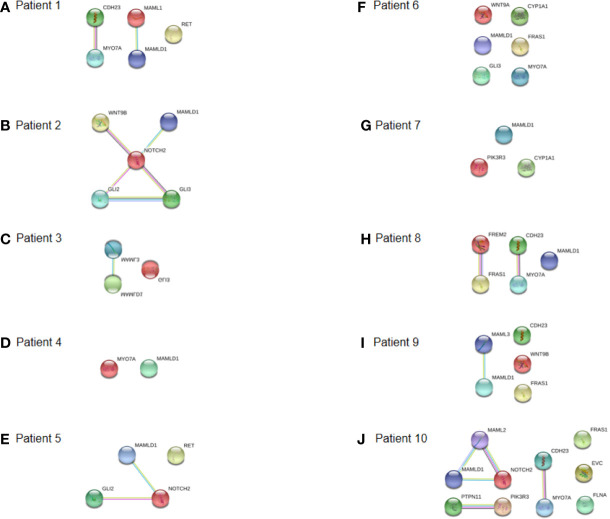
Interaction networks of DSD- and MAMLD1-related genes identified per MAMLD1 individual. **(A)** to **(J)** correspond to the interaction networks per patient. Filled nodes show proteins with known or predicted 3D structure. Empty nodes depict proteins with unknown 3D structure. Candidate genes are underlined. Known interactions correspond to curated databases (turquoise lines) and experimentally determined interactions (pink lines). Predicted interactions correspond to gene neighborhood (green lines), gene fusions (red lines) and gene co-occurrence (blue lines). Other interactions correspond to text mining (yellow lines), co-expression (black lines) and protein homology (violet lines).

The specific interactome of the genes identified in the patients is shown in **Figure 5**. In patients 2, 5, and 10, *MAMLD1* and *MAMLD1*-related genes (*MAML1*, *MAML2*, and *MAML3*) were directly related to *NOTCH2* (**Figures 5B, E, J**). There were two networks each for patients 1 and 8: *CHD23-MYO7A* and *MAML1-MAMLD1* for patient 1 (**Figure 5A**); *CHD23-MYO7A* and *FREM2-FRAS1* for patient 8. In patient 2, *NOTCH2* was shown to play a central role in its associated with the *WNT9B*, *MAMLD1*, and *GLI2/3* network (**Figure 5B**). In patient 10, *PTPN11*, *PIK3R3*, *CDH23*, and *MYO7A* were directly related (**Figure 5J**). The *CHD23-MYO7A* network was identified in three patients (patients 1, 8, and 10).

## Discussion

The genetic architecture of human inheritance has traditionally been divided into two major types. Typically, complex traits exhibit polygenic architectures resulting from the presence of several common variants with low effect, whereas rare traits are usually associated with monogenic determinants with high effect (24). There is growing evidence that suggests that these two classes of phenotypes might not be as biologically distinct as previously considered, and genetic structure of a lineage is present, rather than a dichotomy (25). Mendelian disorders have also been found to be influenced by multiple or common genetic variants (26–28).

Sex development is a highly complex biological event that requires the expression and regulation of a large number of genes with spatial and temporal precision. Although there is considerable information regarding sex development in individuals with monogenic DSDs, the broad spectrum of phenotypes in numerous DSD cases remains less understood. In this study, we investigated the hypothesis that MAMLD1-related DSD may follow an oligogenic mode of inheritance.

All enrolled patients harboured *MAMLD1* variants, and all of them shared a broad spectrum of phenotypes. The most prevalent phenotype was hypospadias and others included cryptorchidism, bifid scrotum, and/or micropenis. The T levels in patients were within the normal range, and were indicative of the mini-puberty stage. An appropriate T response after hCG stimulation was observed in all patients for whom data were available. Additionally, we detected 43 potential disease-causing variants of 18 genes with reported *MAMLD1* interaction. Interactome analysis of identified DSD-related genes was performed to evaluate the possible gene–protein interactions. Using the obtained information, we constructed a genetic map of potential oligogenic hits identified in the patients with 46,XY DSD harbouring heterozygous *MAMLD1*, keeping in mind the existing view of genetic interactions in male sex determination and development. Our findings provide further evidence that individuals with MAMLD1-related 46,XY DSD could harbour two or more variants of known DSD-related genes. The phenotypic outcome might be determined by multiple genes.

A series of studies have been conducted to elucidate the role of oligogenic inheritance in DSDs. A recent study suggested that the expanded DSD phenotypes associated with *NR5A1* mutations resulted from the oligogenic inheritance of other genes related to testicular development, such as *MAP3K1*, *POR*, *CHD7*, and *AKR1C3* (22, 29). Similarly, Eggers et al. observed a storage effect in a cohort of patients with severe hypospadias. In three patients, they observed the oligogenic inheritance of variants of testis development-related genes (*MAP3K1* and *ZFPM2*) in combination with *VUS*. Another patient with severe hypospadias was observed to carry two disease-causing variants of *HSD3B2* and *GNRHR* (30, 31). In addition, among patients with 46,XY DSD of unknown aetiology, five patients were observed to carry a mutation in *AR*, besides carrying other variants in genes encoding proteins participating in androgen action or gonadal development (31). Recently, Flück et al. investigated additional genetic hits in patients with MAMLD1-related DSDs. Using HTS and a custom-tailored algorithm, they identified 55 potentially deleterious genetic variants of 41 additional genes (23). The above information indicates that oligogenic inheritance may contribute to a broader DSD phenotype than previously reported.

In the present study, seven *MYO7A* variants were identified in five patients (patients 1, 3, 6, 8, and 10), five *CDH23* variants were identified in five patients (patients 1, 8, 9, and 10), and five *FRAS1* variants were identified in four patients (patients 6, 8, 9, and 10). Several gene combinations were identified: *MYO7A-CDH23* in three patients, *NOTCH2-GLI2* in two patients, and *MYO7A-FRAS1* in two patients. *MYO7A, CDH23*, and *FRAS1* were the most frequently identified genes, which suggested a shared genetic basis.

Overall, some of the genes identified in the ten patients harbouring *MAMLD1* variants were previously reported to be associated with specific syndromes in patients with genitourinary anomalies: *RET* was shown to be associated with congenital anomalies of the kidney and the urinary tract (CAKUT) syndrome, *EVC* with Ellis-van Creveld syndrome, *FRAS1* and *FREM2* with Fraser syndrome, *PTPN11* with Noonan syndrome, and *WNT9B* with Mayer-Rokitansky-Küster-Hauser syndrome. However, none of the patients enrolled in this study presented with the complete set of phenotypic features typical to these syndromes, possibly because none of these variants induced the complete impairment of gene expression and protein function. On the contrary, advances in sequencing technology have greatly expanded and challenged the validity of the established phenotypes of known syndromes. In this respect, extensive studies on a greater number of cases should be conducted to obtain better evidence.

Interactome analysis was performed to identify DSD-related genes using bioinformatics software to assess possible gene–protein interactions (**Figures 4** and **5**). Overall, a connection was observed among all 18 genes. *MAMLD1* directly connected to *MAML1/2/3* and *NOTCH1/2*. Through *NOTCH1/2*, eight genes (*WNT9A/9B*, *GLI2/3*, *RET*, *FLNA*, *PTPN11*, and *EYA1*) were associated with *MAMLD1*. Flück et al. have reported the interaction network of genes identified in DSD patients with *MAMLD1* variants (23). However, there is the discrepancy between the present and the previous studies in the scheme of the interaction network. The presence of interactions between *GLI2* and *NOTCH2*, *MYO7A*, and *EYA1* have been displayed in the present study, and a known interaction between *WNT9A* and *NOTCH2* disappeared in the present study. This phenomenon may caused by the version difference of STRING software, and the medium confidence, in the present study, the medium confidence was set to 0.4, and when it was set to be lower, the interaction between *WNT9A* and *NOTCH2* were displayed, which means that the screening criteria in our study were higher than the reported article.

The specific interactome of the identified genes in all the patients studied is shown in **Figure 5**. In patients 2, 5, and 10, MAMLD1 and MAMLD1-related genes (*MAML1*, *MAML2*, and *MAML3*) were directly related to *NOTCH2* (**Figures 5B, E, J**). The core network is illustrated in **Figure 4B**, and shows that *NOTCH 1/2* and MAMLD1-related genes constitute the core genes, which was in line with the reported literature (23). NOTCH signalling is a highly conserved signalling pathway and involves the participation of four transmembrane receptors (32). There is growing evidence that mis-regulation of NOTCH signalling may lead to common disorders, ranging from neuropsychiatric to metabolic disorders (33, 34). Furthermore, somatic mutations in genes encoding specific components of the pathway and/or mis-regulation of NOTCH signalling activity have also been linked to oncogenesis and tumour progression in different cancer types (35, 36). The efficient regulation of this pathway was also shown to be essential for the regulation of embryonic development in multiple organ systems, including the gonadal system (37). NOTCH signalling is implicated in Leydig cell differentiation in an inhibitory regulatory manner (37). Further investigations that focus on the functional impacts of each pathogenic mutation will likely provide a better mechanistic understanding of how specific phenotypes may be linked to defects in the NOTCH signalling pathway.

This study has several limitations. The sample size was considerably small to establish a relationship between the observed genotypes and phenotypes, and due to the small sample size, the impact of mutation or variant types had to be excluded from a genotype-phenotype correlation analysis, which reduced the significance of the observations. In addition, functional studies are necessary to further clarify the exact disease-causing effect in patients with 46,XY DSD harbouring MAMLD1 variations; however, when multiple variants are being searched for, which may contribute only partially, this testing method cannot be considered feasible. Therefore, in future studies, we intend to increase the sample size and also extend the follow-up period. Additionally, we intend to employ next-generation statistical analyses of genetic data to identify associations between a group of variants and complex traits in sex development. Moreover, recent improvements in gene editing enabled by advancements in the CRISPR-Cas 9 technology may provide an opportunity for testing the hypotheses on the potential of oligogenic inheritance in DSDs in the near future (38).

In conclusion, we believe our findings provide evidence that individuals with MAMLD1-related 46,XY DSDs could harbour two or more variants of known DSD-related genes. The phenotypic outcomes might be determined by multiple genes. A more extensive study involving other DSD cohorts is necessary to assess whether the genetic variants identified in this study are truly related to DSDs, and that the inclusion of these variants might help establish a better genotype–phenotype correlation.

## Data Availability Statement

The original contributions presented in the study are included in the article, further inquiries can be directed to lilele2006@163.com.

## Ethics Statement

The studies involving human participants were reviewed and approved by the Institutional Medical Ethics Review Board of Beijing Children’s Hospital (ID: 2019-k-156). Written informed consent was obtained from all patients or their legal guardians. Written informed consent to participate in this study was provided by the participants’ legal guardian/next of kin.

## Author Contributions

CG examined and recruited the patients, conceived and designed the study, provided critical comments, and edited the manuscript. LL and FG collected and analysed the data and drafted the manuscript. All authors contributed to the article and approved the submitted version.

## Funding

This study was funded by the Special Fund of the Pediatric Medical Coordinated Development Center of Beijing Hospitals Authority (No.XTYB201808) and the Beijing Natural Science Foundation (No.7204260).

## Conflict of Interest

The authors declare that the research was conducted in the absence of any commercial or financial relationships that could be construed as a potential conflict of interest.
